# (*S*)-Ethyl 1,2,3,9-tetra­hydro­pyrrolo[2,1-*b*]quinazoline-1-carboxyl­ate

**DOI:** 10.1107/S1600536808008908

**Published:** 2008-04-10

**Authors:** Chao Ma, Gui-Jie Du, Yu Tian, Yu Sha, Mao-Sheng Cheng

**Affiliations:** a School of Pharmaceutical Engineering, Shenyang Pharmaceutical University, Mail Box 40, 103 Wenhua Road, Shenhe District, Shenyang 110016, People’s Republic of China; b JinZhou JiuTai Pharmaceutical Co.,Ltd, Taianli, Taihe District, Jinzhou 121012, People’s Republic of China

## Abstract

The title chiral compound, C_14_H_16_N_2_O_2_, was prepared by esterification of (*S*)-1,2,3,9-tetra­hydro­pyrrolo[2,1-*b*]quinazol­in-1-carboxylic acid in the presence of HCl/EtOH. In the mol­ecule, the quinazoline ring is non-planar and exhibits a distorted half-chair conformation, while the five-membered ring shows a typical envelope conformation. Inter­molecular C—H⋯N hydrogen bonding helps to stabilize the crystal structure.

## Related literature

For general background, see: Cheng *et al.* (2006 ▶); Hua *et al.* (2002 ▶).
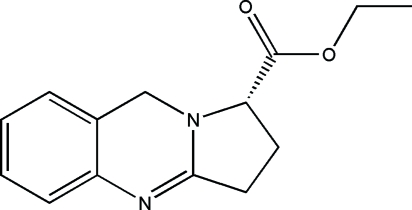


## Experimental

### 

#### Crystal data

C_14_H_16_N_2_O_2_
*M* *_r_* = 244.29
Monoclinic, 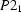

*a* = 6.0545 (8) Å
*b* = 9.1438 (13) Å
*c* = 11.5228 (16) Å
β = 92.905 (2)°
*V* = 637.10 (15) Å^3^
*Z* = 2
Mo *K*α radiation
μ = 0.09 mm^−1^
*T* = 187 (2) K
0.29 × 0.22 × 0.19 mm

#### Data collection

Bruker SMART APEX CCD area-detector diffractometer
Absorption correction: none
3430 measured reflections
1246 independent reflections
1166 reflections with *I* > 2σ(*I*)
*R*_int_ = 0.016

#### Refinement

*R*[*F* ^2^ > 2σ(*F* ^2^)] = 0.031
*wR*(*F* ^2^) = 0.076
*S* = 1.08
1246 reflections
164 parameters
1 restraint
H-atom parameters constrained
Δρ_max_ = 0.11 e Å^−3^
Δρ_min_ = −0.14 e Å^−3^

### 

Data collection: *SMART* (Bruker, 1997 ▶); cell refinement: *SAINT* (Bruker, 1999 ▶); data reduction: *SAINT*; program(s) used to solve structure: *SHELXS97* (Sheldrick, 2008 ▶); program(s) used to refine structure: *SHELXL97* (Sheldrick, 2008 ▶); molecular graphics: *SHELXTL-Plus* (Sheldrick, 2008 ▶); software used to prepare material for publication: *SHELXL97*.

## Supplementary Material

Crystal structure: contains datablocks I, global. DOI: 10.1107/S1600536808008908/xu2409sup1.cif

Structure factors: contains datablocks I. DOI: 10.1107/S1600536808008908/xu2409Isup2.hkl

Additional supplementary materials:  crystallographic information; 3D view; checkCIF report

## Figures and Tables

**Table 1:** Hydrogen-bond geometry (Å, °)

*D*—H⋯*A*	*D*—H	H⋯*A*	*D*⋯*A*	*D*—H⋯*A*
C5—H5⋯N2^i^	0.95	2.59	3.523 (3)	169

Symmetry code: (i) 
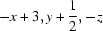
.

**Fig. 1.**
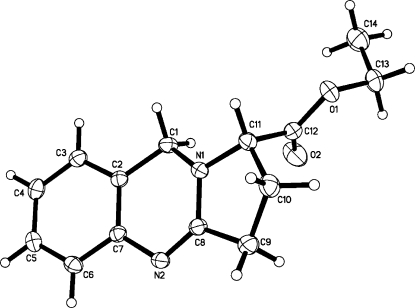
The structure of the title compound, showing 30% probability displacement ellipsoids and the atom-numbering scheme.

**Fig. 2.**
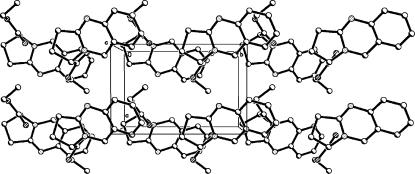
The molecular packing of the title compound.

## supplementary crystallographic information

### Comment

The title chiral compound is a derivate of (*S*)-1,2,3,9-tetrahydro-pyrrolo(2,1 - b)quinazolin-1-carboxylic acid (Linaria acid). Linaria acid is a nature compound, was isolated from the Linaria vulgaris (Hua *et al.*, 2002). Linaria vulgaris is a grassy plant that occurs in northeast China. The plant is used in traditional folk medicine for the treatment of coughs and asthma and as an expectorant. As part of our search on new Linaria acid derivate compounds (Cheng *et al.*, 2006), the title compound is recently synthesized and its crystal structure is reported here.

The molecular structure is shown in Fig. 1. The bond lengths and angles are within normal ranges. The quinazoline moiety is not planar, the central N-heterocyclic ring shows a distorted conformation, with atom N1 and C8 displaced by 0.420 Å and 0.257 Å from the mean plane defined by atoms C1/C2/C7/N2. The five-membered ring adopts an envelope conformation, with atom C10 deviating by 0.443 Å from the plane formed by the other atoms in the ring. Atom C11 of the title molecule is chiral, S configuration was assigned to this atom based on the known chirality of the equivalent atom in the starting material. An intermolecular C—H···N hydrogen bonding (Table 1) helps to stabilize the crystal structure.

### Experimental

A rapid stream of hydrogen chloride was passed for 3 h into absolute ethanol (200 ml) in an icebath. To this solution was added (*S*)-1,2,3,9-tetrahydro-pyrrolo(2,1 - b)quinazolin-1-carboxylic acid (4.32 g, 20 mmol), and this solution was refluxed for 3 h. The ethanol was removed under vacuum. The pure product was obtained through silica gel chromatography (eluant: petroleum ether/ethyl acetate, 1:10). Single crystals suitable for X-ray diffraction were obtained by slow evaporation of a dilute solution of the title compound in ethyl acetate at room temperature.

### Refinement

All H atoms were placed in geometrically idealized positions and constrained to ride on their parent atoms, with C—H = 0.95, 0.99, 0.98 and 1.00 Å for phenyl, methylene, methyl and tertiary H atoms, respectively, with *U*_iso_(H) = *xU*_eq_(C), where *x*=1.5 for methyl H, and *x*=1.2 for all other H atoms. Based on known chirality of the equivalent atom in the starting material, the S chirality at C11 was assigned. Friedel pairs were merged.

### Crystal data

**Table:**

C_14_H_16_N_2_O_2_	*F*_000_ = 260
*M**_r_* = 244.29	*D*_x_ = 1.273 Mg m^−^^3^
Monoclinic, *P*2_1_	Mo *K*α radiation λ = 0.71073 Å
Hall symbol: P 2yb	Cell parameters from 1588 reflections
*a* = 6.0545 (8) Å	θ = 2.9–25.0º
*b* = 9.1438 (13) Å	µ = 0.09 mm^−^^1^
*c* = 11.5228 (16) Å	*T* = 187 (2) K
β = 92.905 (2)º	Block, colorless
*V* = 637.10 (15) Å^3^	0.29 × 0.22 × 0.19 mm
*Z* = 2	

### Data collection

**Table:**

Bruker SMART APEX CCD area-detector diffractometer	1166 reflections with *I* > 2σ(*I*)
Radiation source: fine-focus sealed tube	*R*_int_ = 0.016
Monochromator: graphite	θ_max_ = 25.4º
*T* = 187(2) K	θ_min_ = 1.8º
φ and ω scans	*h* = −7→7
Absorption correction: none	*k* = −11→5
3430 measured reflections	*l* = −13→13
1246 independent reflections	

### Refinement

**Table:**

Refinement on *F*^2^	Secondary atom site location: difference Fourier map
Least-squares matrix: full	Hydrogen site location: inferred from neighbouring sites
*R*[*F*^2^ > 2σ(*F*^2^)] = 0.031	H-atom parameters constrained
*wR*(*F*^2^) = 0.076	*w* = 1/[σ^2^(*F*_o_^2^) + (0.0381*P*)^2^ + 0.082*P*] where *P* = (*F*_o_^2^ + 2*F*_c_^2^)/3
*S* = 1.08	(Δ/σ)_max_ = 0.001
1246 reflections	Δρ_max_ = 0.11 e Å^−^^3^
164 parameters	Δρ_min_ = −0.14 e Å^−^^3^
1 restraint	Extinction correction: none
Primary atom site location: structure-invariant direct methods	

### Special details

**Table:**

Geometry. All e.s.d.'s (except the e.s.d. in the dihedral angle between two l.s. planes) are estimated using the full covariance matrix. The cell e.s.d.'s are taken into account individually in the estimation of e.s.d.'s in distances, angles and torsion angles; correlations between e.s.d.'s in cell parameters are only used when they are defined by crystal symmetry. An approximate (isotropic) treatment of cell e.s.d.'s is used for estimating e.s.d.'s involving l.s. planes.
Refinement. Refinement of *F*^2^ against ALL reflections. The weighted *R*-factor *wR* and goodness of fit *S* are based on *F*^2^, conventional *R*-factors *R* are based on *F*, with *F* set to zero for negative *F*^2^. The threshold expression of *F*^2^ > σ(*F*^2^) is used only for calculating *R*-factors(gt) *etc*. and is not relevant to the choice of reflections for refinement. *R*-factors based on *F*^2^ are statistically about twice as large as those based on *F*, and *R*- factors based on ALL data will be even larger.

### Fractional atomic coordinates and isotropic or equivalent isotropic displacement parameters (Å^2^)

**Table:**

	*x*	*y*	*z*	*U*_iso_*/*U*_eq_	
C1	0.8935 (4)	0.4244 (2)	0.24523 (19)	0.0343 (5)	
H1A	0.9134	0.4636	0.3252	0.041*	
H1B	0.7364	0.4369	0.2188	0.041*	
C2	1.0401 (3)	0.5062 (2)	0.16534 (17)	0.0301 (5)	
C3	0.9824 (4)	0.6432 (3)	0.12309 (18)	0.0360 (5)	
H3	0.8452	0.6850	0.1421	0.043*	
C4	1.1216 (4)	0.7206 (3)	0.05344 (18)	0.0403 (5)	
H4	1.0813	0.8155	0.0263	0.048*	
C5	1.3199 (4)	0.6585 (3)	0.02377 (18)	0.0385 (5)	
H5	1.4157	0.7105	−0.0244	0.046*	
C6	1.3781 (4)	0.5209 (3)	0.06430 (18)	0.0348 (5)	
H6	1.5135	0.4786	0.0430	0.042*	
C7	1.2412 (3)	0.4433 (2)	0.13602 (16)	0.0295 (5)	
C8	1.1581 (3)	0.2248 (3)	0.22061 (16)	0.0309 (5)	
C9	1.1809 (4)	0.0669 (3)	0.2550 (2)	0.0413 (6)	
H9A	1.2678	0.0565	0.3297	0.050*	
H9B	1.2532	0.0098	0.1946	0.050*	
C10	0.9409 (4)	0.0170 (3)	0.2667 (2)	0.0428 (6)	
H10A	0.9307	−0.0560	0.3297	0.051*	
H10B	0.8795	−0.0261	0.1931	0.051*	
C11	0.8183 (3)	0.1590 (3)	0.29605 (17)	0.0351 (5)	
H11	0.6653	0.1587	0.2590	0.042*	
C12	0.8108 (4)	0.1795 (3)	0.42708 (19)	0.0387 (6)	
C13	0.6211 (4)	0.1044 (4)	0.5926 (2)	0.0562 (8)	
H13A	0.7692	0.1147	0.6322	0.067*	
H13B	0.5573	0.0102	0.6169	0.067*	
C14	0.4776 (5)	0.2258 (4)	0.6279 (2)	0.0595 (7)	
H14A	0.3311	0.2158	0.5886	0.089*	
H14B	0.5433	0.3193	0.6063	0.089*	
H14C	0.4636	0.2228	0.7123	0.089*	
N1	0.9521 (3)	0.2705 (2)	0.24410 (14)	0.0315 (4)	
N2	1.3078 (3)	0.3013 (2)	0.17238 (15)	0.0328 (4)	
O1	0.6431 (3)	0.1034 (2)	0.46705 (14)	0.0454 (4)	
O2	0.9396 (3)	0.2508 (3)	0.48572 (14)	0.0652 (6)	

### Atomic displacement parameters (Å^2^)

**Table:**

	*U*^11^	*U*^22^	*U*^33^	*U*^12^	*U*^13^	*U*^23^
C1	0.0276 (11)	0.0351 (13)	0.0405 (11)	0.0037 (10)	0.0048 (9)	−0.0020 (10)
C2	0.0287 (10)	0.0315 (12)	0.0298 (10)	−0.0010 (9)	−0.0006 (8)	−0.0023 (9)
C3	0.0340 (11)	0.0361 (12)	0.0380 (11)	0.0045 (10)	0.0007 (9)	−0.0005 (10)
C4	0.0511 (14)	0.0309 (12)	0.0383 (11)	0.0021 (11)	−0.0026 (10)	0.0044 (11)
C5	0.0450 (12)	0.0389 (13)	0.0316 (11)	−0.0062 (11)	0.0036 (9)	0.0033 (10)
C6	0.0296 (10)	0.0399 (13)	0.0351 (11)	−0.0049 (10)	0.0042 (9)	−0.0039 (11)
C7	0.0276 (10)	0.0320 (12)	0.0285 (10)	0.0003 (9)	−0.0014 (8)	−0.0011 (9)
C8	0.0282 (10)	0.0337 (11)	0.0307 (10)	0.0034 (10)	0.0013 (8)	−0.0002 (9)
C9	0.0376 (12)	0.0370 (14)	0.0495 (14)	0.0034 (11)	0.0055 (10)	0.0055 (10)
C10	0.0451 (14)	0.0366 (13)	0.0471 (13)	−0.0041 (11)	0.0065 (11)	0.0043 (11)
C11	0.0279 (10)	0.0412 (13)	0.0361 (11)	−0.0061 (10)	0.0011 (8)	0.0062 (10)
C12	0.0308 (11)	0.0479 (15)	0.0372 (11)	0.0032 (10)	0.0001 (9)	0.0092 (10)
C13	0.0580 (16)	0.073 (2)	0.0391 (13)	0.0063 (16)	0.0170 (12)	0.0200 (14)
C14	0.0631 (16)	0.0670 (18)	0.0495 (14)	−0.0021 (16)	0.0120 (12)	−0.0016 (15)
N1	0.0274 (9)	0.0328 (11)	0.0347 (9)	0.0008 (8)	0.0050 (7)	0.0042 (8)
N2	0.0260 (9)	0.0347 (11)	0.0380 (9)	0.0017 (8)	0.0046 (7)	0.0019 (8)
O1	0.0442 (9)	0.0514 (11)	0.0418 (9)	−0.0038 (8)	0.0140 (7)	0.0093 (8)
O2	0.0519 (11)	0.1046 (18)	0.0385 (9)	−0.0257 (12)	−0.0036 (8)	0.0003 (11)

### Geometric parameters (Å, °)

**Table:**

C1—N1	1.452 (3)	C9—C10	1.536 (3)
C1—C2	1.509 (3)	C9—H9A	0.9900
C1—H1A	0.9900	C9—H9B	0.9900
C1—H1B	0.9900	C10—C11	1.541 (4)
C2—C3	1.383 (3)	C10—H10A	0.9900
C2—C7	1.403 (3)	C10—H10B	0.9900
C3—C4	1.387 (3)	C11—N1	1.450 (3)
C3—H3	0.9500	C11—C12	1.524 (3)
C4—C5	1.387 (3)	C11—H11	1.0000
C4—H4	0.9500	C12—O2	1.199 (3)
C5—C6	1.381 (3)	C12—O1	1.332 (3)
C5—H5	0.9500	C13—O1	1.459 (3)
C6—C7	1.394 (3)	C13—C14	1.480 (4)
C6—H6	0.9500	C13—H13A	0.9900
C7—N2	1.416 (3)	C13—H13B	0.9900
C8—N2	1.293 (3)	C14—H14A	0.9800
C8—N1	1.355 (3)	C14—H14B	0.9800
C8—C9	1.502 (3)	C14—H14C	0.9800
			
N1—C1—C2	108.86 (17)	C9—C10—C11	103.7 (2)
N1—C1—H1A	109.9	C9—C10—H10A	111.0
C2—C1—H1A	109.9	C11—C10—H10A	111.0
N1—C1—H1B	109.9	C9—C10—H10B	111.0
C2—C1—H1B	109.9	C11—C10—H10B	111.0
H1A—C1—H1B	108.3	H10A—C10—H10B	109.0
C3—C2—C7	119.57 (19)	N1—C11—C12	111.57 (19)
C3—C2—C1	121.18 (19)	N1—C11—C10	102.49 (17)
C7—C2—C1	119.23 (18)	C12—C11—C10	111.05 (18)
C2—C3—C4	121.1 (2)	N1—C11—H11	110.5
C2—C3—H3	119.5	C12—C11—H11	110.5
C4—C3—H3	119.5	C10—C11—H11	110.5
C5—C4—C3	119.5 (2)	O2—C12—O1	125.1 (2)
C5—C4—H4	120.2	O2—C12—C11	125.0 (2)
C3—C4—H4	120.2	O1—C12—C11	109.86 (19)
C6—C5—C4	119.9 (2)	O1—C13—C14	111.2 (2)
C6—C5—H5	120.0	O1—C13—H13A	109.4
C4—C5—H5	120.0	C14—C13—H13A	109.4
C5—C6—C7	121.0 (2)	O1—C13—H13B	109.4
C5—C6—H6	119.5	C14—C13—H13B	109.4
C7—C6—H6	119.5	H13A—C13—H13B	108.0
C6—C7—C2	118.89 (19)	C13—C14—H14A	109.5
C6—C7—N2	118.26 (19)	C13—C14—H14B	109.5
C2—C7—N2	122.80 (18)	H14A—C14—H14B	109.5
N2—C8—N1	126.3 (2)	C13—C14—H14C	109.5
N2—C8—C9	125.2 (2)	H14A—C14—H14C	109.5
N1—C8—C9	108.43 (19)	H14B—C14—H14C	109.5
C8—C9—C10	103.55 (19)	C8—N1—C11	113.89 (18)
C8—C9—H9A	111.0	C8—N1—C1	121.87 (18)
C10—C9—H9A	111.1	C11—N1—C1	122.40 (17)
C8—C9—H9B	111.1	C8—N2—C7	115.34 (18)
C10—C9—H9B	111.1	C12—O1—C13	116.7 (2)
H9A—C9—H9B	109.0		

### Hydrogen-bond geometry (Å, °)

**Table:**

*D*—H···*A*	*D*—H	H···*A*	*D*···*A*	*D*—H···*A*
C5—H5···N2^i^	0.95	2.59	3.523 (3)	169

Symmetry codes: (i) −*x*+3, *y*+1/2, −*z*.

## References

[bb1] Bruker (1997). *SMART* Bruker AXS Inc., Madison, Wisconsin, USA.

[bb2] Bruker (1999). *SAINT* Bruker AXS Inc., Madison, Wisconsin, USA.

[bb3] Cheng, M.-S., Li, Q., Lin, B., Sha, Y., Ren, J.-H., He, Y., Wang, Q.-H., Hua, H.-M. & Kenneth, R. (2006). *Tetrahedron Asymmetry*, **17**, 179–183.

[bb4] Hua, H.-M., Cheng, M.-S., Li, X. & Pei, Y.-H. (2002). *Chem. Pharm. Bull.* **50**, 1393–1394. 10.1248/cpb.50.1393 12372872

[bb5] Sheldrick, G. M. (2008). *Acta Cryst.* A**64**, 112–122. 10.1107/S0108767307043930 18156677

